# Spatial intra-tumour heterogeneity and treatment-induced genomic evolution in oesophageal adenocarcinoma: implications for prognosis and therapy

**DOI:** 10.1186/s13073-024-01362-z

**Published:** 2024-07-17

**Authors:** Sandra Brosda, Lauren G. Aoude, Vanessa F. Bonazzi, Kalpana Patel, James M. Lonie, Clemence J. Belle, Felicity Newell, Lambros T. Koufariotis, Venkateswar Addala, Marjan M. Naeini, AGITG DOCTOR Investigators, John Simes, Euan T. Walpole, Gang T. Mai, David I. Watson, Chris S. Karapetis, Val Gebski, Elizabeth H. Barnes, Martijn Oostendorp, Kate Wilson, Stephen P. Ackland, Jenny Shannon, Gavin Marx, Matthew Burge, Robert Finch, Janine Thomas, Suresh Varma, Louise Nott, John V. Pearson, Lutz Krause, Nicola Waddell, Andrew P. Barbour

**Affiliations:** 1 Frazer Institute, The University of Queensland, 37 Kent Street, Woolloongabba, QLD 4102 Australia; 2 QIMR Berghofer Medical Research Institute, Herston, QLD 4006 Australia; 3 Garvan Institute of Medical Research, Darlinghurst, NSW 2010 Australia; 4 Faculty of Medicine, St Vincent’s Clinical School, University of New South Wales, Sydney, NSW 2052 Australia; 5 Microba Life Sciences, Brisbane, QLD 4000 Australia; 6 Princess Alexandra Hospital, Woolloongabba, QLD 4102 Australia

**Keywords:** Tumour evolution, Whole-genome sequencing, Treatment impact, Oesophageal adenocarcinoma, Genetics

## Abstract

**Background:**

Oesophageal adenocarcinoma (OAC) is a highly heterogeneous cancer with poor survival. Standard curative treatment is chemotherapy with or without radiotherapy followed by oesophagectomy. Genomic heterogeneity is a feature of OAC and has been linked to treatment resistance.

**Methods:**

Whole-genome sequencing data from 59 treatment-naïve and 18 post-treatment samples from 29 OAC patients was analysed. Twenty-seven of these were enrolled in the DOCTOR trial, sponsored by the Australasian Gastro-Intestinal Trials Group. Two biopsies from each treatment-naïve tumour were assessed to define ‘shared’ (between both samples) and ‘private’ (present in one sample) mutations.

**Results:**

Mutational signatures SBS2/13 (APOBEC) and SBS3 (BRCA) were almost exclusively detected in private mutation populations of treatment-naïve tumours. Patients presenting these signatures had significantly worse disease specific survival. Furthermore, mutational signatures associated with platinum-based chemotherapy treatment as well as high platinum enrichment scores were only detected in post-treatment samples. Additionally, clones with high putative neoantigen binding scores were detected in some treatment-naïve samples suggesting immunoediting of clones.

**Conclusions:**

This study demonstrates the high intra-tumour heterogeneity in OAC, as well as indicators for treatment-induced changes during tumour evolution. Intra-tumour heterogeneity remains a problem for successful treatment strategies in OAC.

**Supplementary Information:**

The online version contains supplementary material available at 10.1186/s13073-024-01362-z.

## Background

Oesophageal adenocarcinoma (OAC) is a poor outcome cancer with few treatment options [1]. Over 50% of patients have recurrent disease and all relapses are currently incurable. The standard curative treatment is platinum-based chemotherapy, with or without radiation therapy (RT), followed by oesophagectomy [2]. Immunotherapy has shown benefits in patients with advanced and minimal residual disease in clinical trials (Keynote 590 [3], Checkmate 577 [4]). However, many patients do not benefit or have irreversible treatment-related side effects.

OAC tumours have complex genomes characterised by a high tumour mutation burden (TMB) [5] and high clonal diversity resulting in intra-tumour heterogeneity (ITH) [6, 7]. Defects in DNA repair pathways and treatment-related mechanisms contribute to the high number of accumulated mutations during tumour progression [8, 9]. Whole-genome sequencing (WGS) studies have identified mutational signatures related to DNA damage repair, high mutation load and age in OAC, as well as signature 17 as a hallmark signature of treatment-naïve OAC [5, 10–12].

ITH has been identified as a major challenge for cancer therapies [13, 14]. It is often responsible for the development of drug-resistant clones and therefore treatment failure [15] and relapse after surgery [16]. In OAC, high ITH has been associated with poor response to neoadjuvant treatment [6]. While ITH has generally been linked to poor prognosis in many solid cancer types, such as breast, kidney and prostate cancers [17, 18], it has also been associated with improved patient prognosis in high-risk neuroblastoma [19].

The clonal tumour composition changes over time and is considered to be both cause and consequence of genomic heterogeneity [20, 21]. It remains a challenge for precision medicine and emerging treatment options like immunotherapy [22]. Recent TRACERx studies in lung cancer have shown the potential of multi-region and multi-timepoint sampling to identify which and when subclones are most likely to seed metastases [23, 24]. They have also shown that certain subclonal events such as subclonal expansion and whole genome doubling results in shorter disease-free survival [16]. In OAC, few studies have assessed ITH and tumour evolution. One study used whole-exome sequencing data from matched single-region samples before and after chemotherapy for 30 cases and showed higher mutation burden pre-treatment (‘genetic bottleneck’) for treatment responders [7]. Another study compared WGS data from treatment-naïve and chemotherapy treated tumours using both matched and unmatched single-region samples per time point and reported few genomic changes after neoadjuvant treatment [10].

Understanding ITH and assessing mutational processes which occur across samples or with treatment will be highly beneficial to precision medicine in OAC. We designed a prospective study with multi-region sampling both before and after chemo(radio)therapy. Using WGS, 77 tumour samples from 29 OAC patients were assessed to characterise ITH, both spatially and temporally in response to therapy. Based on the available multi-region samples, we assessed ITH between samples from the same patient, where shared mutations were found in all assessed samples and private mutations were found in only one sample. We further compared mutations that were unique to pre- and post-treatment biopsies. Twenty-seven patients were treated on the DOCTOR clinical trial (Australian New Zealand Clinical Trials Registry ACTRN12609000665235, sponsored by the Australasian Gastro-Intestinal Trials Group [AGITG]) [25].

## Methods

### Patient cohort and sample collection

A total of 77 primary tumour samples from 29 patients with OAC were included in this whole-genome sequencing (WGS) study. Written informed consent has been obtained from all patients. Twenty-seven patients were enrolled in the clinical phase II AGITG DOCTOR trial (Australian New Zealand Clinical Trials Registry, ACTRN12609000665235 [25]). Trial patients received one cycle of neoadjuvant chemotherapy (cisplatin and 5-fluorouracil [CF]) and treatment response was assessed on day 14. Responders continued with a second cycle of CF. Patients showing poor response to the initial rounds of chemotherapy (non-responders) were randomised into two additional cycles of docetaxel treatment (DCF), ± 45 Gy radiotherapy (RT). Two additional patients were recruited through the Upper GastroIntestinal Unit at the Princess Alexandra Hospital, Brisbane, Australia, by the Cancer Evolution Biobank. Non-trial patients received platinum-based neoadjuvant treatment with radiotherapy (carboplatin + paclitaxel + RT) or went directly to surgery and contributed only multi-region treatment-naïve samples.

Clinical data including stage, tumour size and treatment was collected for all patients (Table 1, Additional file 1: Table S1). Disease specific survival (DSS) was determined as the time from endoscopy until death from disease and was censored at 60 months. No perioperative deaths were recorded.

**Table 1:** Summarised clinical characteristics of the study cohort

	Total cohort (*n* = 29)
DOCTOR trial patients	*N* = 27
Non-trial patients	*N* = 2
Age at diagnosis	Mean: 59.45 y; median: 60 y; range: 38–71 y
Gender	
Male	28 (96.6%)
Female	1 (3.4%)
Clinical stage (cTNM) (8th AJCC edition)	
IB	1 (3.4%)
IIB	6 (20.7%)
III	21 (72.4%)
IVA	1 (3.4%)
Treatment	
Pre-op chemo	18 (62.1%)
Pre-op CXRT	10 (34.5%)
Direct to surgery	1 (3.4%)
Follow-up from endoscopy	
Alive	12 (37.9%): mean: 55.67 mo; median: 60 mo; range: 44–60 mo
Dead	17 (62.1%): mean: 19.35 mo; median: 13 mo; range: 3–39 mo
Pathological stage (ypTNM) (8th AJCC edition)	
0	2 (6.9%)
I	7 (24.1%)
II	3 (10.3%)
III	9 (31%)
IVA	6 (20.7%)
IVB	2 (6.9%)
Pathological response	Mean: 41.2%; median: 20% range: 0–100%
Mandard score	
> 90% regression	6 (20.7%)
50–90% regression	2 (6.9%)
< 50% regression	13 (44.8%)
Unknown	8 (27.6%)

*AJCC* American Joint Committee on Cancer, *mo* Months, *y* Years

Tumour samples were collected at endoscopy (treatment-naïve; *n* = 59) and at surgery (after neoadjuvant chemo(radio)therapy; *n* = 18) and stored in RNAlater. At each time point, multi-region samples were taken approximately 20 mm apart. For each patient, a peripheral blood or normal oesophageal tissue sample was collected at endoscopy as the matched normal control. DNA was extracted from the buffy coat using the Qiagen Flexigene kit according to manufacturer’s protocol. DNA was extracted from tumour and normal tissue samples using the Qiagen AllPrep DNA/RNA mini kit according to standard protocol (Qiagen, Germany). All tumour samples had sufficient tumour content (cellularity) for WGS (median 69.9%; range 38–94%; Additional file 1: Table S2) based on qpure estimations from Illumina SNP array data [26].

### Whole-genome sequencing and mutation calling

A total of 106 gDNA samples (*n* = 77 tumour, *n* = 29 normal) underwent library preparation (TruSeq PCR free Library kit) and WGS on an HiSeq Xten (150 bp paired-end; Illumina, San Diego, CA, USA). Targeted sequencing depth was 30 × for normal and 60 × for tumour samples. After sequencing, Cutadapt (v1.9) [27] was applied to trim adaptors. BWA-MEM (v0.7.15) [28] and SAMtools (v1.9) [29] were used to map the reads to human genome assembly version GRCh37, marking duplicated reads with Picard MarkDuplicates (v2.8.15) [30].

Single nucleotide variants (SNVs) were identified using the latest version of the analysis pipeline created for Australia’s participation in the International Cancer Genome Consortium. It uses output from qSNP (v2.1.4) [31] and GATK HaplotypeCaller (v4.0.4.0) [32] as raw inputs for a heuristics-based system that re-interrogates the BAM files, calls variants, assigns confidence to the calls, assesses mutational consequence and annotates variants. The output is a series of VCFs (and MAF files for somatic calls) that contain variant calls at different levels of confidence. Variants underwent Ensembl gene annotation using SnpEff [33].

Mutations called in both treatment-naïve samples were classified as ‘shared’, and mutations called in only one sample as ‘private’. When post-treatment samples were included, ‘shared’ mutations were called in all available pre- and post-treatment samples of a patient. Mutations called in any treatment-naïve, but no post-treatment sample were classified as ‘unique pre’ and mutations called in any post-treatment, but no treatment-naïve sample were defined as ‘unique post’.

For OESO_0001, three treatment-naïve samples were available and used for the comparison of pre- and post-treatment samples. For the assessment of spatial heterogeneity in treatment-naïve OACs, the two samples that were most similar in tumour cellularity (47.4% T1 and 51.01% T2) were selected for this patient (Additional file 1: Table S2).

### Neoantigen prediction and DAI score

Class I HLA genotypes were computed for tumour-normal pairs of WGS using Optitype (v1.3.1) [34] with default parameters. Neoantigen prediction was performed using pVAC-Seq (v4.0.10) [35] pipeline with default parameters and binding affinity was estimated using NetMHCpan (v4.0) [36]. Variants were annotated for wild-type and mutant peptide sequences through variant effect predictor (VEP; v86) from ENSEMBL. Epitopes with binding affinity inhibitory concentration (IC50) ≤ 500 nM were considered putative neoantigens that bind to HLA alleles [37].

The differential agretopicity index (DAI) was calculated for each putative neoantigen by subtracting its predicted binding affinity from the corresponding wild-type peptide binding affinity [37, 38].

### Mutational signatures

Mutational signatures were assigned using the non-negative matrix factorisation method described by Alexandrov et al. [39]. The contribution of each COSMIC single base substitution (SBS) signature (v3.3) was estimated using a quadratic programming approach from the R package SignatureEstimation (v1.0.0) [40]. Only signatures SBS1/2/3/5/8/13/17a/17b/18/20/26/36/39/40/44/93 were present at a minimum of 10% in at least one sample and used for further analyses. Mutation populations (mutations classified as shared/private or assigned to subclone C1/C2/etc.) were then used to estimate the prevalence of this set of signatures. To prevent over-fitting, signatures < 5% were removed for each population and mutations were reassigned to the remaining signatures. To identify treatment-related changes, signatures SBS31 and SBS35 were included in the analysis of pre- and post-treatment samples. To identify mutational signatures in tumour clones (e.g. C1), only mutations unique to the clone were considered to avoid overlapping information.

### Platinum enrichment

In gDNA, platinum-based treatment introduces C > A substitutions in a CpC context [41]. To measure the changes induced in the post-treatment samples (*n* = 18), a platinum signature enrichment odds ratio was calculated. It detects enrichment for C > A (or G > T) mutations in a CpC (or GpG) context using 41-base regions centred on the mutated cytosine (or guanine) [6].

### Clonal composition analysis

PyClone-VI was used to estimate the clonal composition of each sample using mutation and copy number data [42]. For each patient, the mutation calls from each sample (T1/T2/etc.) were combined and read counts for each sample for each mutation were collated in a mutation pileup file using qbasepileup [43]. Copy number was determined from the WGS data using ascatNGS (v4.0.1) [44] and added for each mutation. PyClone-VI was configured to allow up to 40 clusters (clones) and 100 random restarts. Identified clones containing less than 1% of a tumour’s mutations were excluded from further analyses. Clonal evolution trees were generated through manual review of the PyClone-VI outputs. Clonal prevalences were averaged between the available samples of each timepoint to establish pre- and post-treatment tumour profiles and visualised in R using the *fishplot* package (v0.5.1) [45].

### Statistical analysis and data visualisation

All statistical analyses and figure generation were performed in R (v4.1.2) and the significance level was set to 0.05. Pairwise comparisons of continuous variables used the Student’s *t*-test and false discovery rate (fdr) to correct for multiple testing. Contingency tables were analysed using Fisher’s exact tests and significant differences between multiple groups were tested using Kruskal-Wallis tests. Survival analyses were performed using Kaplan-Meier curves (log-rank test) of the R packages *survival* (v3.4.0) [46, 47] and *survminer* (v0.4.9) [48]. Oncoplots were generated using the *maftools* package (v2.8.5) [49], contingency tables were visualised using the *ggstatsplot* package (v0.11.0) [50], and all other figures were prepared using the *ggplot2* package (v3.4.0) [51].

## Results

### Patient cohort

**Fig. 1:**
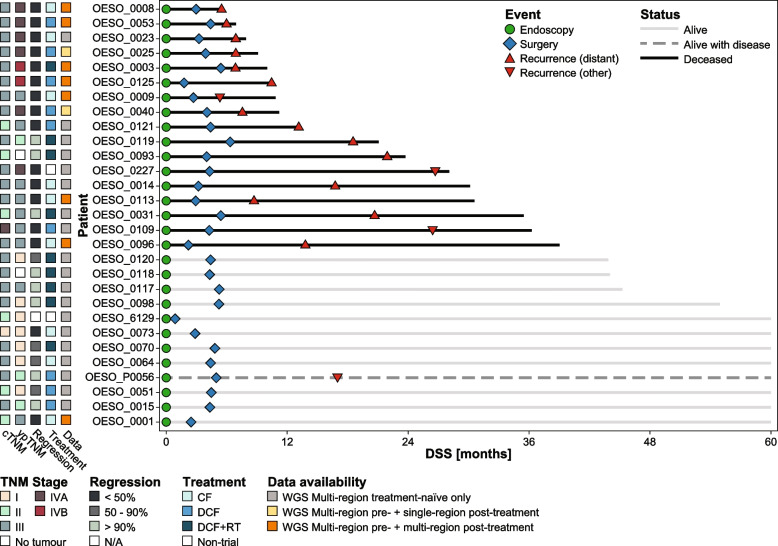
Swimmer plot of patient cohort. Visualisation of the disease specific survival (DSS) and status for each patient as well as clinical events. DSS has been censored after 60 months. Clinical stage (cTNM), pathological stage (ypTNM), tumour regression at surgery, allocated treatments and data availability per patient are displayed on the left. CF, cisplatin and 5-fluorouracil; DCF, CF and docetaxel; RT, 45 Gy radiotherapy; WGS, whole-genome sequencing

To assess ITH in OAC, WGS analysis was performed on 77 primary tumour samples from 29 OAC patients, 27 of which were treated on the AGITG DOCTOR trial [25]. Most patients (72%) presented with stage III disease (Table 1). The analysis included 59 treatment-naïve samples taken at endoscopy and 18 samples taken at surgery after treatment with neoadjuvant chemo(radio)therapy (Fig. 1, Table 1). The median age of disease onset was 60 years (range 38–71 years). The median DSS was 35 months (range 6–60 months). Median follow-up for survivors was 60 months (range 44–60 months; Fig. 1, Table 1).

### Intra-tumour heterogeneity in treatment-naïve OAC

To understand ITH and clonal composition of OAC, multi-region sequencing was performed whereby 2 spatially distinct regions of 29 treatment-naïve tumours were assessed.

**Fig. 2:**
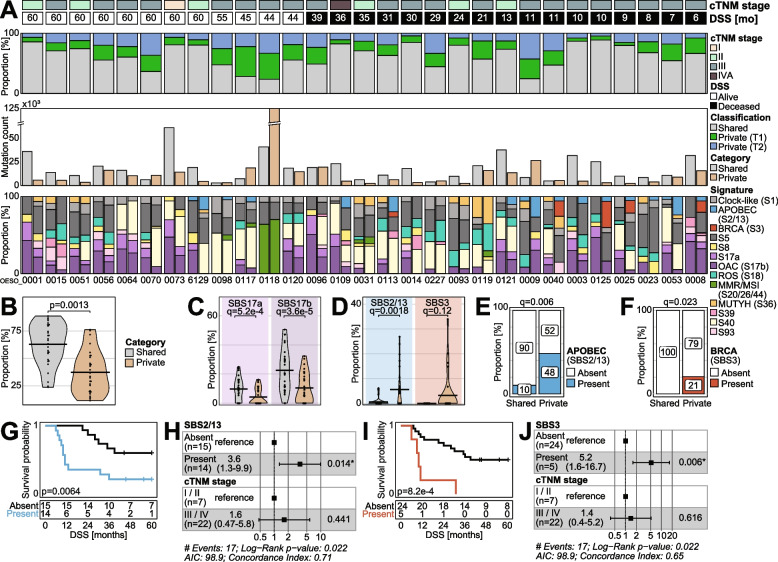
Mutational signatures in the shared and private mutation populations of the multi-region treatment-naïve samples. Shared mutations are shared across both treatment-naïve samples; private mutations were only detected in one sample. **A** The top panel of bar plots shows the proportion of shared (grey) and private mutations. The private mutations are split into private to sample T1 (green) and private to sample T2 (blue). The middle panel shows the absolute number of mutations used in the mutational signature analysis. The bottom panel of stacked bar plots shows the mutational signatures identified in the shared (first bar) and private (second bar) mutation populations of each tumour. Signatures SBS2 and SBS13 as well as SBS20, SBS26 and SBS44 are combined to an APOBEC and an MMR/MSI signature, respectively. Patients are ordered by survival status and time. **B**–**D** Violin plots of **B** the proportions of shared and private mutations per tumour, **C** the proportions of signatures SBS17a and SBS17b in the shared and private mutation populations and **D** signature SBS2/13 (APOBEC) and SBS3 (BRCA) proportions in the shared and private mutation populations. *q*-values represent the fdr corrected *p*-values. **E**–**F** Stacked bar plots showing the contingency table of the presence of the **E** APOBEC associated signatures (SBS2/13) and **F** BRCA associated signature (SBS3) in the shared and private mutation populations. **G**–**H** Disease specific survival (DSS) of patients stratified by presence of mutational signatures SBS2/13 (APOBEC) in the private mutations **G** in a Kaplan-Meier plot (log-rank test) and **H** a forest plot of the associated hazard ratios after adjusting for clinical cTNM stage (cox regression). **I**–**J** DSS for patients with signature SBS3 (BRCA) present or absent in the private mutation populations in **I** a Kaplan-Meier plot and **J** a forest plot of the hazard ratios after adjustment for clinical stage. SBS, single base substitution; OAC, oesophageal adenocarcinoma; ROS, reactive oxygen species; MMR, mismatch repair; MSI, microsatellite instability

On average, treatment-naïve OAC samples harboured 26,618.76 mutations (median 21,362.5; range 4665–139,985; Additional file 1: Table S2) resulting in a mean TMB (mutations/Mb) of 9.32 (median 7.51; range 1.62–48.8; Additional file 1: Table S2). In 21 cases, most mutations were found in both treatment-naïve samples (shared). In the remaining 8 cases (OESO_0070, OESO_0098, OESO_0117, OESO_0118, OESO_0096, OESO_0227, OESO_0009, OESO_0040), the analysed samples showed higher numbers of private mutations (present in only one treatment-naïve sample; Fig. 2A). Overall, samples from the same tumour showed similar mutation numbers and proportions of shared and private mutations (Additional file 1: Table S2). The number of private mutations was not associated with tumour cellularity (*R* = −0.16; data not shown). The average proportion of shared mutations across the cohort was 62.82% (median 66.48%; range 23.72–88.65%; Fig. 2A). There was a significantly higher proportion of shared mutations than private mutations (*p* = 0.001; paired *t*-test; Fig. 2B). However, no association between proportion of shared/private mutations and DSS was observed (*p* = 0.32; cox regression).

### Mutational processes in shared and private mutations in treatment-naïve OAC are associated with survival

Mutational signatures associated with shared mutations were compared to private mutations (Fig. 2A). The shared and private mutations of OESO_0118 were dominated by mutations assigned to multiple signatures associated with defective DNA mismatch repair (MMR) and microsatellite instability (MSI; SBS20, SBS26 and SBS44). This tumour contained a shared somatic pathogenic nonsense mutation in *MSH2* (NM_000251.2:c.970C > T; p.Gln324*) [52]. It is a shared event which arises early and continues to drive mutations in this tumour resulting in significant ITH, high overall SNV burden and high numbers of private mutations (Fig. 2A). MMR/MSI signatures were also found in private mutations of OESO_0098, OESO_0117 and OESO_0119 and in both shared and private mutations of OESO_0031. However, no non-synonymous mutations in MMR/MSI pathway genes [53] were found in these cases. OESO_0119 displayed the highest proportions of SBS36 across both shared and private mutations. This signature has been associated with defective base excision repair due to *MUTYH* mutations [54]. It aligns with a shared pathogenic somatic variant and loss of heterozygosity in *MUTYH* (NM_001128425.2:c.1213C > T; p.Pro405Leu) in this tumour [52]. It indicates an early event, which still drives active mutational processes in each distinct sample.

Significantly higher proportions of signatures 17a/b were detected among the shared mutations (*q* < 5.2e − 4; paired *t*-test; Fig. 2C; Additional file 1: Table S3). Signature 17 is a hallmark signature of OAC containing predominantly T > G substitutions in a CTT context. It is considered to be associated with gastric acid reflux [5, 8, 55] and has been detected in the precancerous disease Barrett’s oesophagus [12]. It can also occur later after 5-fluorouracil treatment [56]. In contrast, significantly higher proportions of SBS2/13 (*q* = 0.0018; paired *t*-test) were found in the private mutation populations (Fig. 2D). Additionally, proportions of SBS3 were significantly higher (*p* = 0.042; paired *t*-test), however did not remain significant after correcting for multiple testing (*q* = 0.12). This suggests that these mutational processes occur later in these tumours. Signatures SBS2 and SBS13 are attributed to AID/APOBEC activity [57], while SBS3 has been associated with defective homologous recombination DNA damage repair (HR-DDR) with a strong association to somatic and germline *BRCA1/2* mutations in other cancers [58]. Notably, in the five tumours with detected SBS3 (OESO_0113, OESO_0040, OESO_0125, OESO_0025 and OESO_0008), only private mutations were assigned to the BRCA signature (SBS3), but no non-synonymous mutations in *BRCA1/2* were found. However, other genes from the HR-DDR pathway were found to harbour private mutations in two of these cases. A private missense mutation in *ATM* was found in OESO_0025 (NM_000051:c.3174G > A; p.Trp1058*) and a private non-synonymous missense variant in *BARD1* (NM_000465:c.2017G > C; p.Asp673His) was found in OESO_0113. Furthermore, SBS3 was always found in conjunction with APOBEC signatures (SBS2/13). We dichotomised signatures that showed significantly higher proportions in the private mutation populations into present/absent. The increased presence of signatures SBS2/13 and SBS3 in the private mutation populations remained statistically significant (*p* = 0.003/*q* = 0.006 and *p* = 0.023/*q* = 0.023, respectively; Fisher’s exact test/fdr corrected; Fig. 2E/F) and were then assessed for associations with DSS.

Patients with APOBEC signatures (SBS2/13) present in their private mutation populations had significantly worse DSS (*p* = 0.0064; log-rank; Fig. 2G). The association remained significant after adjusting for clinical cTNM stage (*p* = 0.014; cox regression; Fig. 2H). Furthermore, the five patients with signature SBS3 present in their private mutation populations showed worse survival outcomes (*p* = 8.2e − 4; log-rank; Fig. 2I), which remained significant after adjustment for clinical stage (*p* = 0.006; cox regression; Fig. 2J).

### Recurrently mutated genes

The most frequently mutated gene in OAC is *TP53*, with approximately 80% of patients harbouring a mutation in this gene [5, 8, 12]. *TP53* mutations in our cohort were assessed to determine whether they were shared or private events. As expected, shared non-synonymous *TP53* mutations were detected in 24 (83%) tumours indicating *TP53* mutations are driver events in these tumours. Twelve (50%) of these shared *TP53* variants were pathogenic or likely-pathogenic variants (ClinVar; Additional file 1: Table S4). Other genes reported as cancer related in COSMIC (v98) showing high proportions of shared non-synonymous mutations were *MUC16* (10 patients), *FAT4* (6 patients) and *CDKN2A* (7 patients; Additional file 2: Fig. S1A). Cancer-related genes harboured more shared (mean 1.01; range 0–24) than private non-synonymous mutations (mean 0.83; range 0–5); however, the statistical test did not reach significance (*p* = 0.15; paired *t*-test; Additional file 2: Fig. S1B). Genes without non-synonymous mutations were removed from the analysis.

### Clonal composition

**Fig. 3:**
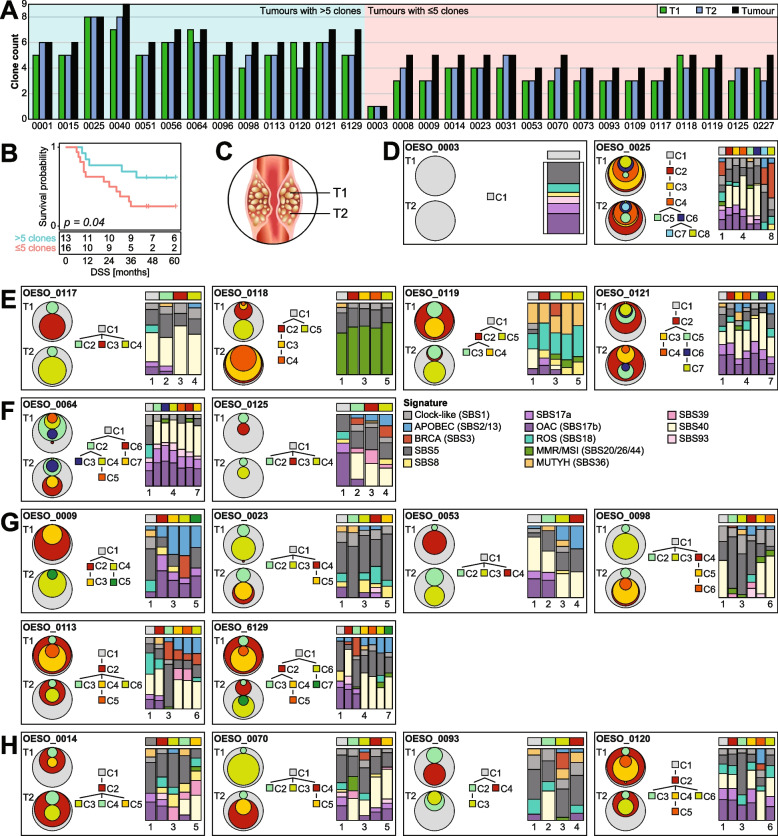
Clonal compositions detected in multi-region treatment-naïve samples. **A** Bar plot of number of clones detected per sample (T1/T2) and per tumour (union of clones identified in T1 and T2). The patients are grouped by tumour clone number above average (5 clones). **B** Kaplan-Meier plot for disease specific survival (DSS; log-rank test) stratified by high (> 5) and low (≤ 5) clone numbers. **C** Schematic map of sample collection. **D**–**H** Circle plots, clonal evolution trees and mutational signature bar plots. The circle plots visualise the clonal composition and proportions of identified clones in each sample. Each box contains the patient ID, two circle plots (one per sample), a phylogenetic tree showing the relationship between clones, and the mutational signatures per clone per patient. Tumours with **D** the same clones detected in both samples, **E** similar mutational signature profile between all clones, and **F** different mutational signatures in the founder clone (C1) and the subclones (C2-7) of the treatment-naïve samples. **G** APOBEC signatures detected in subclonal mutation populations were traced back to individual clones. **H** Additional cases of APOBEC signatures in unique subclones

To define genomic diversity in OAC, an assessment of the clonal composition of the treatment-naïve biopsies of 29 tumours was performed using PyClone-VI. On average treatment-naïve tumours contained 5 clones (range 1–9; Fig. 3A). High mutation rate was not associated with high clone numbers (Additional file 2: Fig. S2A).

Next, the association between the clonal composition of the tumour and patient survival was assessed. A high number of clones was associated with improved DSS (*p* = 0.04; Fig. 3B), however did not reach statistical significance after adjusting for clinical stage (*p* = 0.057; Additional file 2: Fig. S2B). No significant associations between clone numbers and cellularity or clinical features (age, stage and tumour size) were found (Additional file 2: Fig. S2C–H).

Two tumours displayed a similar clonal structure between their two spatial treatment-naïve samples, with OESO_0003 containing a single clone present in both samples (C1) and OESO_0025 with the same 8 clones present in both samples (Fig. 3D). In fourteen cases, even though spatially distant biopsies harboured the same number of clones (e.g. 3 clones in each biopsy from OESO_0117), the clonal composition of each biopsy was different (Fig. 3E–H, Additional file 2: Fig. S3). The remaining tumours displayed different number of clones between the two spatially distant samples. All but two cases (OESO_0003 and OESO_0025) had at least one clone that was unique to one of the two samples (e.g. C3 in T1 and C4 in T2 in OESO_0117). Had only one of the biopsies been sequenced, the subclones unique to the other sample would have been missed (Fig. 3E–H, Additional file 2: Fig. S3).

The mutations assigned to each clone were used to investigate the mutational signatures per clone. On average, 5334 mutations were assigned to a clone (range 69–69,572; Additional file 1: Table S5). Interestingly, for tumours OESO_0117, OESO_0118, OESO_0119 and OESO_0121 (Fig. 3E), the mutational signatures were similar between all clones (C1–C7). In contrast, OESO_0064 and OESO_0125 showed different signature profiles between the founder clone (C1) and the subclones (C2-7) indicating different timings for different mutational processes (Fig. 3F).

The APOBEC signatures (SBS2/13) detected in subclonal mutation populations (Fig. 2A) could be traced back to individual clones. For example for OESO_0009, all four subclones that are not shared between the two samples exhibit proportions of signatures SBS2/13 (C2-5) with a small proportion also detected in C1 (Fig. 3G). Four cases (OESO_0014, OESO_0070, OESO_0093 and OESO_0120; Fig. 3H) presented APOBEC signatures in unique subclones (e.g. in C3 in OESO_0014); however, the signal was not strong enough to be detected when all subclonal mutations of the tumour were analysed together in Fig. 2A.

### Treatment impacts tumour evolution

To assess the effect of platinum-based neoadjuvant treatment on OAC evolution, multi-region WGS data derived from both pre- and post-treatment samples for 10 patients with minimal pathological response to neoadjuvant therapy was analysed (*n* = 39 samples; Fig. 1). Somatic mutations were classified as present in all samples (clonal), or exclusively present in treatment-naïve (unique pre) or post-treatment samples (unique post).

**Fig. 4:**
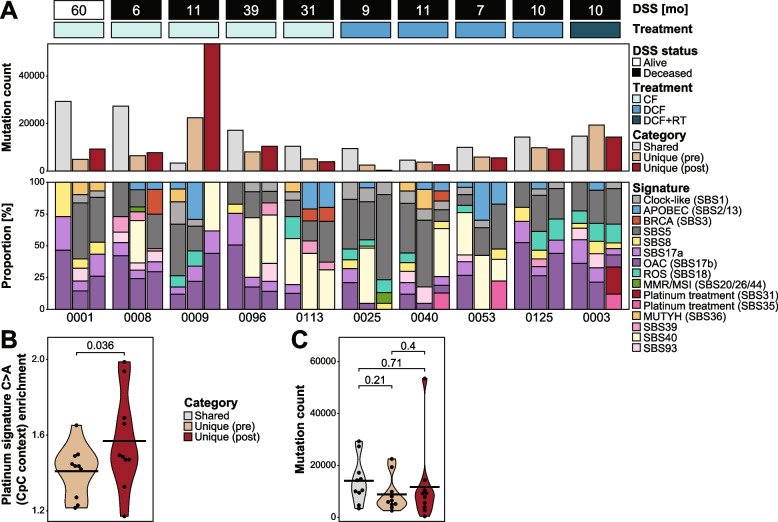
Mutational signatures in multi-region and multi-timepoint samples. **A** Mutation populations were divided into shared (shared across all samples), unique pre (present in any treatment-naïve sample but no post-treatment sample) and unique post (present in any post-treatment sample but no treatment-naïve sample). The top panel shows bar plots of the number of mutations, while the bottom panel shows the proportion of mutational signatures detected in each mutation population as stacked bar plots. **B**–**C** Violin plots of **B** the platinum enrichment scores in the unique pre- and post-treatment mutation populations and **C** the number of mutations in each subset

To obtain a better understanding of underlying mechanisms driving mutagenesis pre- and post-treatment, we assigned the mutations unique to each time point to mutational signatures. Signatures associated with platinum-based chemotherapy treatment (SBS31 and SBS35) [59] were detected in the unique post-treatment mutations of three patients (OESO_0040, OESO_0053 and OESO_0003) and were absent from unique pre-treatment mutations (Fig. 4A). Notably, all three patients received DCF treatment (OESO_0003 with additional radiotherapy). Furthermore, platinum enrichment scores examining platinum-treatment-induced C > A substitutions in a CpC context [6] were significantly increased in the unique post-treatment mutations of these samples (*p* = 0.036, paired *t*-test; Fig. 4B). The tumours OESO_0003 and OESO_0009 were the only two cases with a higher number of unique mutations than shared mutations. Across the cohort, there were no significant differences in the number of mutations unique to pre- and post-treatment samples (*p* = 0.4; Fig. 4C).

### Immunoediting in OAC

We further investigated subclonal putative neoantigens that were unique to pre- and post-treatment samples and used the differential agretopicity index (DAI) as a marker of immunologically relevant peptides. Neoantigens with a high DAI have been shown to be more immunogenic [37, 38]. There was no statistically significant difference between the DAI scores of putative neoantigens between the two groups (*p* = 0.89; *t*-test; data not shown). Noteworthy, the median DAI score among the unique post putative neoantigens was 90.33, while the median for the unique pre putative neoantigens was 138.89. While this difference did not reach statistically significant difference (*p* = 0.5; Kruskal-Wallis), it indicates that there might be pressure during tumour evolution that selects for less immunogenic tumour cell populations in some, but not all cases.

### Tumour evolution under treatment and immune pressure

**Fig. 5:**
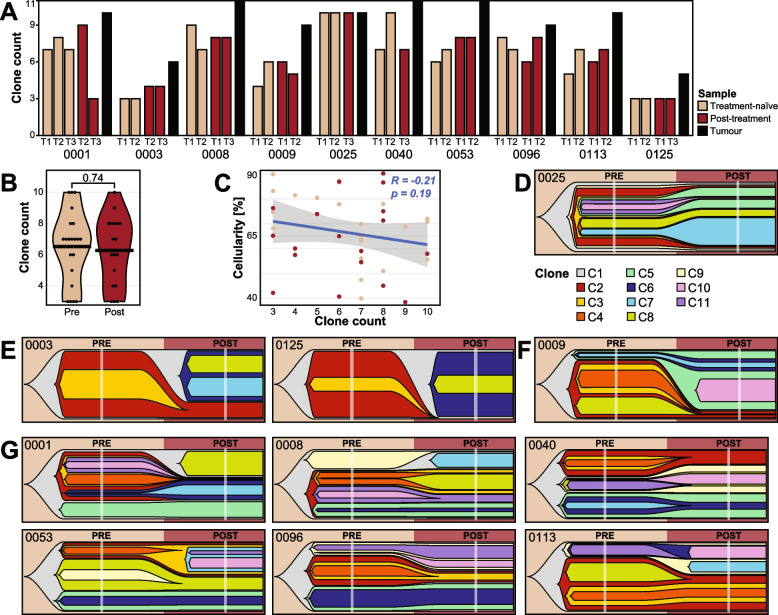
Clonal evolution over time. **A** Number of clones detected in each sample and the tumour (union of clones identified in all samples of the tumour). **B** Violin plot of clone numbers in pre- and post-treatment samples. **C** Scatter plot of clone numbers and tumour cellularity per sample including linear model and Spearman’s rank correlation. **D**–**G** Fishplots visualising the clonal evolution. Each fishplot shows the average clone proportions in the pre- and post-treatment tumour. **D** Tumour case showing a relatively stable clonal composition over time. **E**–**F** A clonal sweep was detected either **E** by itself or **F** in addition to clonal expansion. **G** Cases showing that treatment pressure can affect different parts of the tumour differently, leading to complex evolution patterns. The clone IDs are used to distinguish clones of each patient but cannot be used for comparison between patients

Resolving the clonal composition using pre- and post-treatment samples revealed increased clone numbers with an average of 9 clones per tumour (range 5–11; Fig. 5A) compared to the analysis of only treatment-naïve samples with an average of 5 clones per tumour (range 1–9; Fig. 3). There was no significant difference of clone numbers detected in pre- and post-treatment samples of the same tumour (*p* = 0.74; *t*-test; Fig. 5B). The number of clones was not associated with tumour cellularity (*p* = 0.19; Spearman’s rank correlation; Fig. 5C). Furthermore, adding the matched post-treatment samples lead to higher resolution to detect more subclonal events across all samples. It enabled better separation of clones present in multiple samples (e.g. C1 in OESO_0003 split into C1/2/3; Fig. 3, Additional file 2: Fig. S4).

Various patterns of tumour evolution were observed in the changes of clonal composition between the pre- and post-treatment tumours. Pre-treatment tumours were on average 5.3 cm long (range 3–9 cm) and on average 4.32 cm (range 2.5–7.5 cm) post-treatment (Additional file 1: Table S1). The clonal composition of OESO_0025 appears relatively stable between the pre- and post-treatment samples with no new clones detected after treatment (Fig. 5D). The two cases OESO_0003 and OESO_0125 showed an almost complete clonal sweep after treatment (Fig. 5E). Subclones detected in the post-treatment samples were not detected before treatment (e.g. C6/7/8 in OESO_0003). An almost complete clonal sweep after treatment (C2/3/4/8) in combination with expansion of a subclone unique to one treatment-naïve sample (C5) was found in OESO_0009 (Fig. 5F). Most cases displayed a complex evolution pattern with clones shared between time points as well as timepoint specific clones (Fig. 5G). Furthermore, ITH in the post-treatment tumour suggested that different parts of the tumour responded differently to treatment pressure (e.g. OESO_0001; Additional file 2: Fig. S4).

**Fig. 6:**
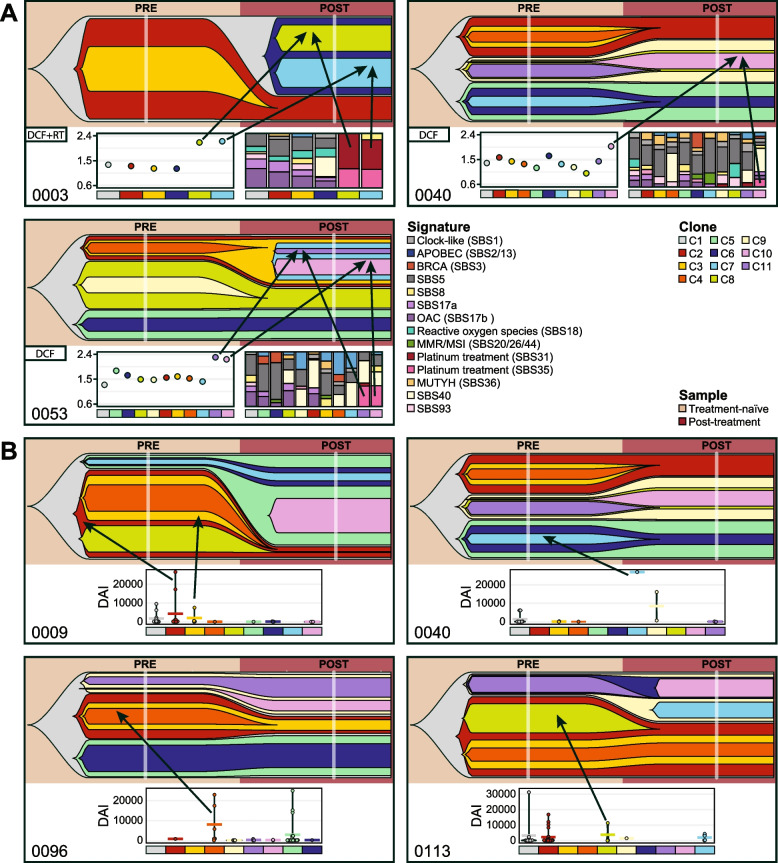
Treatment impact on tumour evolution. Each box contains the fishplot of the clonal composition of each pre- and post-treatment tumour. Below each fishplot are **A** the platinum enrichment score and mutational signatures for each individual clone and **B** the differential agretopicity index (DAI) scores of the predicted neoantigens found in each clone. The clones and their corresponding scores are colour-matched per patient. The crossbars indicate the mean per group

In three cases (OESO_0003, OESO_0040 and OESO_0053), clones newly detected in post-treatment samples could be associated with platinum-based treatment based on increased platinum enrichment scores and platinum-related mutational signatures (Fig. 6A). This suggests that in some cases treatment can induce changes to the tumour genome.

Clonal selection may occur in some cases through immunoediting, with immune-mediated killing of immunogenic clones through detection of tumour neoantigens. This has recently been reported in pancreatic and lung cancer with high-quality neoantigens promoting the most robust immune response [60]. Thus, to investigate clones detected in treatment-naïve samples that were absent from post-treatment samples, the DAI for each putative neoantigen was included as a measure of immunogenicity for each individual clone. Examples of subclones with high DAI putative neoantigens that were not detected after treatment were found in four cases (OESO_0009, OESO_0040, OESO_0096, OESO_0113; Fig. 6B; Additional file 1: Table S6). Thus, in patients with residual tumour after neoadjuvant therapy (i.e. poor responders), immunoediting might take place, but appears limited in extent.

## Discussion

This study focussed on the clonal composition and evolution of OAC using WGS from multi-region and multi-timepoint samples. A total of 77 primary OAC samples (59 treatment-naïve and 18 treated samples) from 29 patients were analysed emphasising the challenge of ITH. Multi-region analyses along with identifying subclones can identify clonal features amenable to targeted therapies enabling the removal of the whole tumour.

ITH was observed at a spatial level in treatment-naïve samples from the same tumour. Most tumours (72%) showed higher proportions of shared than private mutations with no link to patient prognosis. Shared mutations found in all samples of a tumour represent somatic events that occurred early in its tumorigenesis (e.g. *TP53* mutations). On the contrary, private mutations, present in a subset of tumour samples, are the result of late events that can lead to subclonal diversification and tumour progression [61]. Notably, in most cases the number of private mutations unique to each of the two treatment-naïve samples was similar, suggesting equal mutation pressure across the tumour regions. *TP53* was the most mutated gene in our study, supporting previous OAC genomics studies [5, 8, 12]. The mutations harboured in *TP53* and other known cancer-related genes were predominantly shared mutations indicating involvement in the early stages of OAC tumorigenesis. Almost half of the shared non-synonymous *TP53* mutations were recorded as (likely-) pathogenic in ClinVar highlighting its role in OAC development.

Investigating the differences between shared and private mutations of 29 treatment-naïve OAC cases indicated different underlying processes causing early (shared) and late (private) somatic events. Mutational signatures SBS17a/b were significantly enriched in the shared mutation populations indicative of involvement in the early stages of tumorigenesis. Signature 17 has been associated with gastric reflux in treatment-naïve OAC and gastric cancers and has been labelled as a hallmark signature of treatment-naïve OAC [5, 10–12]. It has also been associated with 5-fluorouracil treatment in other cancer types indicating different mutational processes can induce similar signatures [56]. Mutational signatures SBS2/13, attributed to AID/APOBEC activity [57], were enriched in private mutation populations in our cohort and their presence was linked with worse patient survival. The presence of an APOBEC signature in predominantly late-stage OAC samples had been associated with impaired overall patient survival in a previous study which included samples from this study in a single-region analysis of a larger cohort [52]. Our study, however, extends those results linking the presence of APOBEC signatures to patient survival. The APOBEC family of enzymes has been suggested to play a role in tumour development and to promote intra-tumour heterogeneity [61, 62] and targeted therapy resistance in non-small cell lung cancer [63, 64]. APOBEC signatures have also been found in private breast cancer mutations [65]. This suggests that APOBEC cytosine deaminases induce late (private) events in the tumorigenesis of OAC through ‘episodic mutagenesis’ and could be a treatment target [66].

Very similar observations were made for SBS3, a signature linked to defective HR-DDR and *BRCA1/2* mutations, which was exclusively detected in private mutations that also showed evidence of APOBEC signatures. In our cohort, SBS3 occurs late (private) and appears to be HR-DDR like, but not *BRCA1/2* driven, since no private mutations were found in these genes. Two tumours harboured a private non-synonymous mutation in the HR-DDR pathway genes *ATM* and *BARD1*, respectively.

Clonal composition analysis revealed an average of 5 clones per treatment-naïve tumour and patients with highly heterogeneous tumours (> 5 clones) displayed improved DSS. While high heterogeneity has been associated with poor clinical prognosis in many other solid cancer types, such as breast, kidney, brain (lower grade glioma) and prostate cancers [17, 18], OAC seems to follow high-risk neuroblastoma where patients with ITH showed improved survival [19].

Extrinsic factors like chemotherapy as well as intrinsic factors such as the patient’s immune microenvironment put subclonal cell populations under evolutionary pressure to utilise the capacity of malignant cells to adapt and develop resistance to treatment [67]. This leads to increased ITH with a mixture of treatment-sensitive and -resistant subclones impacting treatment response. Clonal composition analysis of matched treatment-naïve and post-treatment samples revealed patterns of clonal sweep with or without clonal expansion following treatment in three cases with minimal pathological response. This is similar to previous reports of ‘bottlenecks’ during tumour evolution and treatment pressure in OAC [7]. Chemotherapy-induced changes to the genome were further detected in three post-treatment tumours after three cycles of chemotherapy. Clones unique to the post-treatment samples exhibited mutational signatures associated with platinum-based chemotherapy (SBS31/35) as well as increased platinum enrichment scores confirming results from previous studies [6, 10]. These three tumours failed to respond to the initial round of CF treatment and received two additional cycles with DCF with or without RT according to the clinical trial protocol. It is noteworthy that the platinum signature was not observed in tumours treated with 2 cycles of CF alone.

Clones present in treatment-naïve but not post-treatment samples were detected. We utilised the concept that immune recognition should be improved for neoantigens with higher peptide self-dissimilarity [37] by assessing the DAI of putative neoantigens within these clones. DAI has been shown to correlate with survival and immune infiltration in both lung cancer and melanoma patients [38]. In four cases, putative neoantigens within treatment-naïve clones displayed high DAI and these clones were not observed in post-treatment samples. This suggests that the immunogenic neoantigens within subclones might have led to their immune-mediated elimination as described in pancreatic cancer [60]. These findings provide insights into tumour-specific immune responses and the associated clonal dynamics during cancer treatment, however need to be validated in functional studies.

In summary, we investigated WGS data from multi-region and multi-timepoint OAC samples. We highlighted the potential role of APOBEC and HR-DDR during late tumour evolution. These subclonal driver processes may portend worse survival but may be targetable. Due to the limited sample size of this study, these findings need to be validated in a larger cohort to determine the clinical impact of the described observations. We further showed evidence of treatment-induced genomic changes including the rise of new clones in patients with minimal pathological response. These changes are restricted to a subset of tumours.

## Conclusions

To improve survival and treatment response rates for OAC patients, innovative approaches are required to develop personalised treatments, involving chemotherapy, targeted and immune-based treatments and companion diagnostic methods for selecting optimal treatment regiments. Targeted treatment based on single-sample analysis of a highly heterogeneous cancer type such as OAC remains challenging [24]. Identified targets may only affect certain subclones, rather than the entire tumour. To develop more effective and less toxic treatment approaches, it is crucial to understand the genomic heterogeneity both between patients and within tumours, and its impact on treatment response.

### Supplementary Information

Additional file 1: Table S1. Clinical information for the cohort. NSR, no sign of recurrence; OG, oesophago-gastric. Table S2. Sample information including estimated tumour cellularity, mutation count and tumour mutation burden (TMB; mutations/Mb). Table S3. Differences in signature proportions between shared and private mutation populations. Comparisons between continuous values used Student’s *t*-test and Fisher’s exact test for dichotomised data. *p*-values were corrected for multiple testing through fdr correction. Table S4. Detected clonal ClinVar variants in *TP53* across the cohort. Table S5. Number of mutations assigned to individual clones. Table S6. Putative neoantigens identified in clones present in pre-treatment but not post-treatment samples.

Additional file 2: Fig. S1. Oncoplot of somatic variants in known cancer-related genes detected in treatment-naïve samples. A Mutations detected in shared and private populations for each tumour. B Violin plots showing the number of shared and private non-synonymous mutations in the COSMIC cancer genes. Crossbars indicate the mean. Fig. S2. Clone counts in association to patient and sample features. A Scatter plot of number of mutations and number of clones. B Forest plot of hazard ratios for disease specific overall survival (DSS) stratified by high (>5) and low (≤5) clone numbers corrected for stage (cox regression). C–F Scatter plots showing the relationship between number of clones and C tumour cellularity, D age, E tumour length at endoscopy and F tumour size at surgery. G–H Violin plots of the number of clones stratified by G the clinical cTNM and H the pathological ypTNM stage. Fig. S3. Clonal compositions of multi-region treatment-naïve samples. Each box contains the circle plots, clonal evolution trees and mutational signature bar plots for each patient. The circle plots visualise the clonal composition and proportions of identified clones in each sample while the phylogenetic trees show the relationship between clones. Fig. S4. Clonal composition of multi-region and multi-timepoint samples. Each box contains the circle plots for each sample collected from the same tumour. The proportions and relationship of the clones are shown in the circle plots. The clone IDs are patient specific and cannot be used to compare clones between patients.

## Data Availability

The whole-genome sequencing data presented in this study is available in the European Genome-phenome Archive, study accession number EGAS00001002864, data set ID EGAD00001015373 (https://ega-archive.org/datasets/EGAD00001015373) [68]. The sequence data are generated from patient samples and therefore are available under restricted access. Data access can be granted via the EGA with completion of an institute data transfer agreement, and data will be available for a defined time period once access has been granted. All major analysis code is available on GitHub (https://github.com/SOG-Lab/OAC_IntraTumourHeterogeneity) [69].

## Abbreviations

AGITG: Australasian Gastro-Intestinal Trials Group
CF: Cisplatin and 5-fluorouracil
DAI: Differential agretopicity index
DCF: Docetaxel, cisplatin, 5-fluorouracil
DSS: Disease specific survival
fdr: False discovery rate
HR-DDR: Homologous recombination-based DNA damage repair
ITH: Intra-tumour heterogeneity
MMR: Mismatch repair
MSI: Microsatellite instability
OAC: Oesophageal adenocarcinoma
RT: Radiation therapy
SBS: Single base substitution
SNV: Single nucleotide variant
TMB: Tumour mutation burden
WGS: Whole-genome sequencing
