# Supplementary material for: Spatial intra-tumour heterogeneity and treatment-induced genomic evolution in oesophageal adenocarcinoma: implications for prognosis and therapy
Source: Genome Med. 2024 Jul 17;16:90. doi: 10.1186/s13073-024-01362-z (PMC11253399; doi:10.1186/s13073-024-01362-z)
Supplement: Supplementary file 2 — Additional file 2: Fig. S1. Oncoplot of somatic variants in known cancer-related genes detected in treatment-naïve samples. A Mutations detected in shared and private populations for each tumour. B Violin plots showing the number of shared and private non-synonymous mutations in the COSMIC cancer genes. Crossbars indicate the mean. Fig. S2. Clone counts in association to patient and sample features. A Scatter plot of number of mutations and number of clones. B Forest plot of hazard ratios for disease specific overall survival (DSS) stratified by high (>5) and low (≤5) clone numbers corrected for stage (cox regression). C–F Scatter plots showing the relationship between number of clones and C tumour cellularity, D age, E tumour length at endoscopy and F tumour size at surgery. G–H Violin plots of the number of clones stratified by G the clinical cTNM and H the pathological ypTNM stage. Fig. S3. Clonal compositions of multi-region treatment-naïve samples. Each box contains the circle plots, clonal evolution trees and mutational signature bar plots for each patient. The circle plots visualise the clonal composition and proportions of identified clones in each sample while the phylogenetic trees show the relationship between clones. Fig. S4. Clonal composition of multi-region and multi-timepoint samples. Each box contains the circle plots for each sample collected from the same tumour. The proportions and relationship of the clones are shown in the circle plots. The clone IDs are patient specific and cannot be used to compare clones between patients. [file 13073_2024_1362_MOESM2_ESM.pdf]

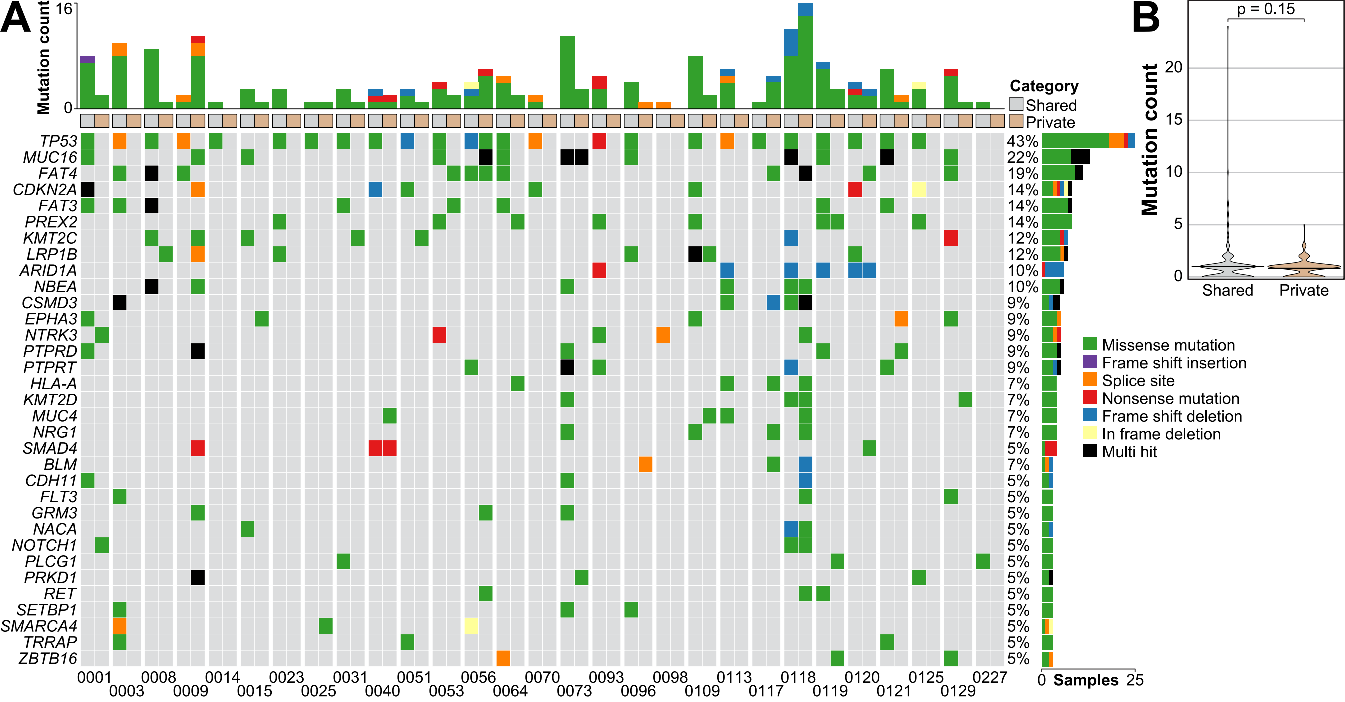

Fig. S1. Oncoplot of somatic variants in known cancer related genes detected in treatment-naïve samples. A. Mutations detected in shared and private populations for each tumour. B. Violin plots showing the number of shared and private non-synonymous mutations in the COSMIC cancer genes. Crossbars indicate the mean.

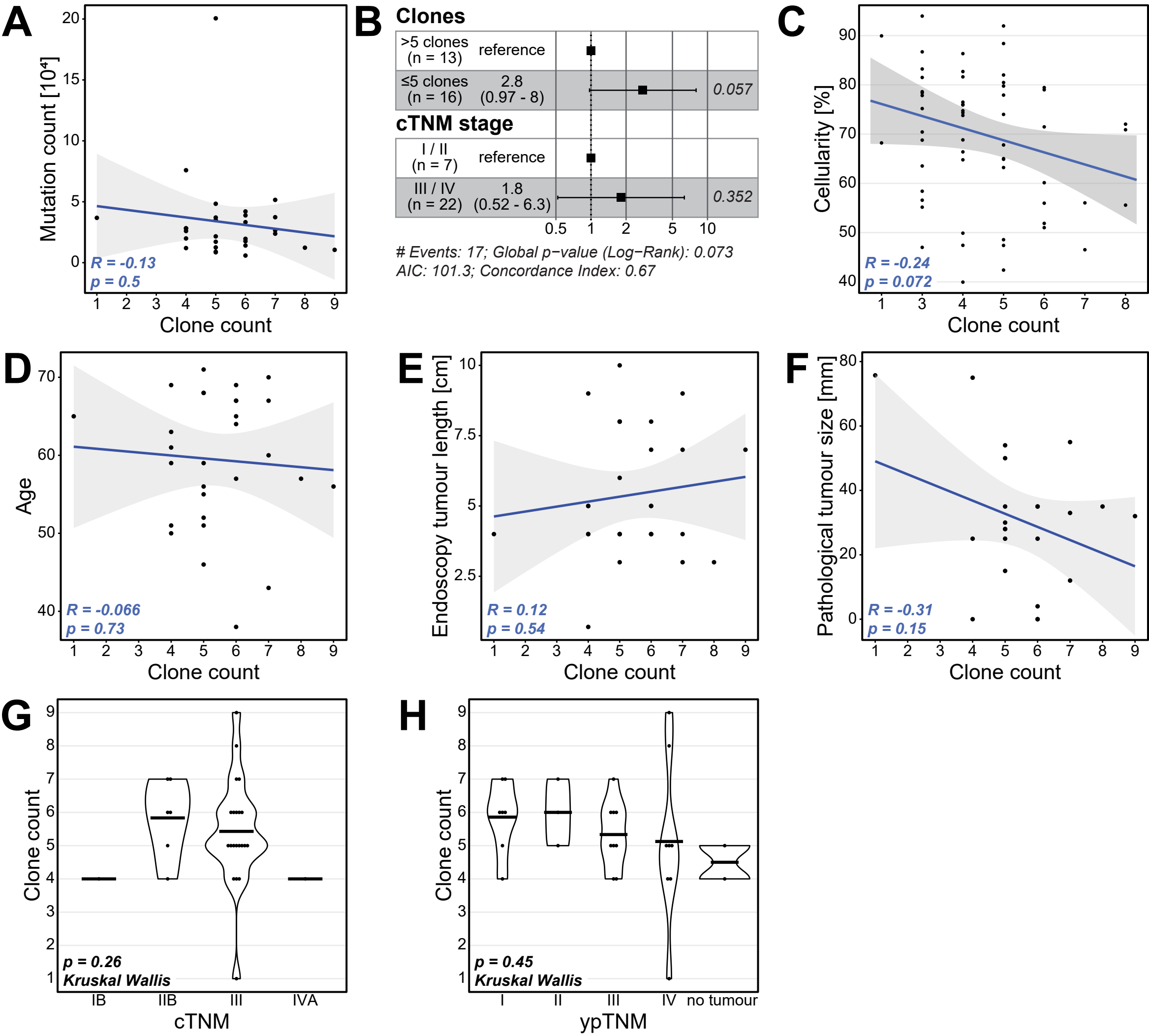

Fig. S2. Clone counts in association to patient and sample features. A. Scatter plot of number of mutations and number of clones. B. Forest plot of hazard ratios for disease specific overall survival (DSS) stratified by high (>5) and low (≤5) clone numbers corrected for stage (cos-regression). C-F. Scatter plots showing the relationship between number of clones and C. tumour cellularity, D. age, E. tumour length at endoscopy and F. tumour size at surgery. G-H. Violin plots of the number of clones stratified by G. the clinical cTNM and H. the pathological ypTNM stage.

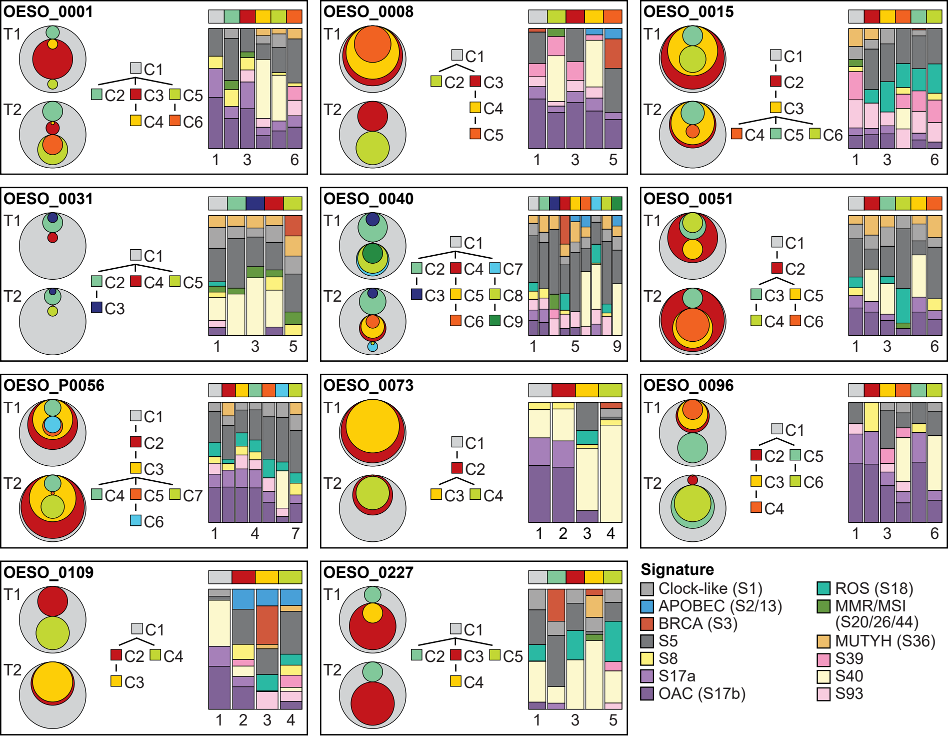

Fig. S3. Clonal compositions of multi-region treatment-naïve samples. Each box contains the circle plots, clonal evolution trees and mutational signature bar plots for each patient. The circle plots visualise the clonal composition and proportions of identified clones in each sample while the phylogenetic trees show the relationship between clones.

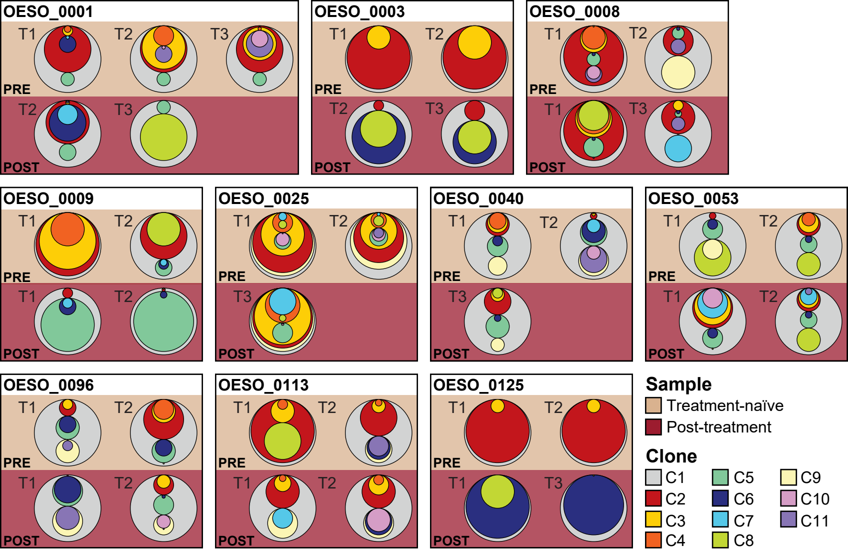

Fig. S4. Clonal composition of multi-region and multi-timepoint samples. Each box contains the circle plots for each sample collected from the same tumour. The proportions and relationship of the clones are shown in the circle plots. The clone IDs are patient specific and cannot be used to compare clones between patients.
